# DNA methylation changes in response to neoadjuvant chemotherapy are associated with breast cancer survival

**DOI:** 10.1186/s13058-022-01537-9

**Published:** 2022-06-24

**Authors:** Christine Aaserød Pedersen, Maria Dung Cao, Thomas Fleischer, Morten B. Rye, Stian Knappskog, Hans Petter Eikesdal, Per Eystein Lønning, Jörg Tost, Vessela N. Kristensen, May-Britt Tessem, Guro F. Giskeødegård, Tone F. Bathen

**Affiliations:** 1 Department of Circulation and Medical Imaging, NTNU - Norwegian University of Science and Technology, Trondheim, Norway; 2 Department of Nursing, Health and Laboratory Science, Østfold University College, Halden, Norway; 3 Department of Cancer Genetics, Institute for Cancer Research, Oslo University Hospital, Oslo, Norway; 4 Clinic of Surgery, St. Olavs Hospital, Trondheim University Hospital, Trondheim, Norway; 5 Department of Clinical and Molecular Medicine, NTNU – Norwegian University of Science and Technology, Trondheim, Norway; 6 Clinic of Laboratory Medicine, St. Olavs Hospital, Trondheim University Hospital, Trondheim, Norway; 7 BioCore - Bioinformatics Core Facility, NTNU – Norwegian University of Science and Technology, Trondheim, Norway; 8 K.G. Jebsen Centre for Genome-Directed Cancer Therapy, Department of Clinical Science, University of Bergen, Bergen, Norway; 9 Department of Oncology, Haukeland University Hospital, Bergen, Norway; 10 Laboratory for Epigenetics and Environment, Centre National de Recherche en Génomique Humaine, CEA – Institut de Biologie François Jacob, Université Paris Saclay, 91000 Evry, France; 11 Department of Medical Genetics, Institute of Clinical Medicine, Oslo University Hospital, Oslo, Norway; 12 K.G. Jebsen Center for Genetic Epidemiology, Department of Public Health, and Nursing, NTNU - Norwegian University of Science and Technology, Trondheim, Norway; 13 Department of Radiology and Nuclear Medicine, St. Olavs Hospital, Trondheim University Hospital, Trondheim, Norway

**Keywords:** DNA methylation, Locally advanced breast cancer, Survival, Treatment response, Breast cancer, Neoadjuvant chemotherapy, Chemotherapy

## Abstract

**Background:**

Locally advanced breast cancer is a heterogeneous disease with respect to response to neoadjuvant chemotherapy (NACT) and survival. It is currently not possible to accurately predict who will benefit from the specific types of NACT. DNA methylation is an epigenetic mechanism known to play an important role in regulating gene expression and may serve as a biomarker for treatment response and survival. We investigated the potential role of DNA methylation as a prognostic marker for long-term survival (> 5 years) after NACT in breast cancer.

**Methods:**

DNA methylation profiles of pre-treatment (*n* = 55) and post-treatment (*n* = 75) biopsies from 83 women with locally advanced breast cancer were investigated using the Illumina HumanMethylation450 BeadChip. The patients received neoadjuvant treatment with epirubicin and/or paclitaxel. Linear mixed models were used to associate DNA methylation to treatment response and survival based on clinical response to NACT (partial response or stable disease) and 5-year survival, respectively. LASSO regression was performed to identify a risk score based on the statistically significant methylation sites and Kaplan–Meier curve analysis was used to estimate survival probabilities using ten years of survival follow-up data. The risk score developed in our discovery cohort was validated in an independent validation cohort consisting of paired pre-treatment and post-treatment biopsies from 85 women with locally advanced breast cancer. Patients included in the validation cohort were treated with either doxorubicin or 5-FU and mitomycin NACT.

**Results:**

DNA methylation patterns changed from before to after NACT in 5-year survivors, while no significant changes were observed in non-survivors or related to treatment response. DNA methylation changes included an overall loss of methylation at CpG islands and gain of methylation in non-CpG islands, and these changes affected genes linked to transcription factor activity, cell adhesion and immune functions. A risk score was developed based on four methylation sites which successfully predicted long-term survival in our cohort (*p* = 0.0034) and in an independent validation cohort (*p* = 0.049).

**Conclusion:**

Our results demonstrate that DNA methylation patterns in breast tumors change in response to NACT. These changes in DNA methylation show potential as prognostic biomarkers for breast cancer survival.

**Supplementary Information:**

The online version contains supplementary material available at 10.1186/s13058-022-01537-9.

## Background

Locally advanced breast cancer is a heterogeneous disease with varying outcomes and different responses to neoadjuvant chemotherapy (NACT), depending on breast cancer subtype. Neoadjuvant treatment has become a standard of care for locally advanced breast cancer, offering the benefit of downstaging the disease prior to surgery and the elimination of disseminated cancer cells to improve survival [1, 2]. To improve personalized treatment and avoid late effects from unnecessary treatment, it is important to develop novel predictive and prognostic biomarkers for patient stratification based on response to NACT and patient survival.

DNA methylation is an epigenetic mechanism that regulates gene expression and chromatin structure. It influences gene expression in a complex manner; for example, promoter CpG island hypermethylation can repress gene transcription, while gene body hypomethylation can increase transcription [3, 4]; however, promoter methylation could also be associated with increased transcription [5, 6]. Aberrant methylation patterns can be detected early in cancer development [7] and have been shown to be important for development and progression in breast cancer and other malignancies [8–11].

Today, the molecular subgrouping of breast cancer [12] as well as axillary lymph node status, tumor size, HER2 overexpression, histopathological grade and hormone receptor status are used to assess patients' risk and to decide treatment options according to national clinical guidelines [13]. However, there is still substantial heterogeneity within the subgroups in respect to response to NACT and survival [8, 9, 14–16]. The added informational value of DNA methylation patterns may contribute to the identification of patients who will respond to treatment and those who will have a more aggressive course of disease.

Several studies have explored how changes in gene expression and metabolite profiles during NACT correlate with treatment response and survival in breast cancer patients [17, 18], while less is known about how DNA methylation changes in response to NACT. A recent study reported differential DNA methylation in whole blood following chemotherapy in breast cancer patients [19], but the changes were not investigated in relation to treatment response or survival. Differential DNA methylation in tumor biopsies has been shown to predict treatment response in breast cancer biopsies [20–22]. For instance, Klajic et al. [20] showed that DNA methylation of cell cycle-related genes changed differently in responders and non-responders during NACT. A study by Luo et al. [23] demonstrated changes in DNA methylation heterogeneity in response to NACT. DNA methylation patterns of pre-treatment biopsies have also been used to predict survival in a doxorubicin-treated cohort by Dejeux et al. [24]. Changes in DNA methylation can influence both treatment response and survival and are therefore important for developing new therapeutic targets and prognostic markers.

Therapy resistance is a major challenge in cancer treatment, and increasing evidence suggests that exposure to chemotherapy may drive drug resistance through silencing and activation of genes caused by methylation [25–28]. In this study, we assess the methylation patterns before and after NACT to determine how NACT affect tumor DNA methylation and investigate the predictive and prognostic potential of treatment-induced changes in DNA methylation patterns.

## Materials and methods

### Patients and treatment protocol

The inclusion criteria and treatment protocol for the EpiTax trial have been reported previously [29, 30]. In brief, the female breast cancer patients (*n* = 83) included in this study were recruited from a larger open-label multicenter study where patients were randomly allocated to receive neoadjuvant anthracycline (epirubicin, 90 mg/m^2^) or taxane (paclitaxel, 200 mg/m^2^) monotherapy. The trial was conducted from 1997 to 2003. Chemotherapy was administered every third week for four cycles. In case of suboptimal treatment response evaluated by the UICC system [31], treatment was switched to the opposite regimen (epirubicin or paclitaxel). All patients received postoperative radiotherapy, and for estrogen receptor positive disease (> 10% staining cells), adjuvant endocrine treatment was given according to national guidelines from the Norwegian Breast Cancer Group applicable at the time. The study was approved by The Regional Committee for Medical and Health Research Ethics (273/96-82.96), Norwegian Health Region III), and informed written consent was obtained from all patients.

### Treatment response and survival

Treatment response was evaluated clinically following guidelines by the UICC system applicable at the time of patient recruitment [31]. Tumor sizes were calculated based on caliper measurements (the product of the two largest tumor diameters) prior to NACT treatment and after completed treatment. Survival follow-up data were collected more than ten years after patient inclusion. The patients in the present study were drawn from two response groups: (1) partial response (≥ 50% reduction in tumor size after treatment) and (2) stable disease (< 50% reduction and ≤ 25% increase in tumor size after treatment). To evaluate breast cancer survival, the patients were classified into two groups: (1) survivors (patients surviving 5 years or more after diagnosis) and (2) non-survivors (breast cancer-specific death within 5 years after diagnosis). Patient and tumor characteristics of the survival groups are shown in Table 1.

**Table 1:** Patient and tumor characteristics of included breast cancer patients undergoing NACT treatment

	5-year survivors, *n* = 59*	5-year non-survivors, *n* = 24*
Age, median (IQR)		
Years	51.5 (44.4 to 56.9)	48.1 (43.9 to 56.8)
Tumor stage, *n* (%)		
IIB	21 (35.6)	7 (29.2)
IIIA	24 (40.7)	12 (50.0)
IIIB	11 (18.6)	3 (12.5)
IV	3 (5.1)	2 (8.3)
Intrinsic subtype, *n* (%)		
Basal	5 (9.3)	4 (20.0)
HER2 enriched	11 (20.4)	5 (25.0)
Luminal A	12 (22.2)	2 (10.0)
Luminal B	18 (33.3)	4 (20.0)
Normal-like	8 (14.8)	5 (25.0)
Treatment response, *n* (%)		
Partial response	41 (69.5)	11 (45.8)
Stable disease	18 (30.5)	13 (54.2)
Treatment, *n* (%)		
Epirubicin	25 (42.4)	8 (33.3)
Paclitaxel	24 (40.7)	6 (25.0)
EpiTax**	10 (16.9)	10 (41.7)

*Survivors, *n* = 59 (24 patients with pre- or post-treatment samples, 35 patients with paired samples). Non-survivors, *n* = 24 (12 patients with pre- or post-treatment samples, 12 patients with paired samples)

**EpiTax: epirubicin followed by paclitaxel or paclitaxel followed by epirubicin

### Sample handling prior to DNA extraction

Tissue biopsies were taken before commencing NACT and after treatment during surgical removal of the tumor. Biopsies were immediately snap-frozen after removal and stored in liquid nitrogen. One piece of the pre-treatment tumor biopsy was used for the assessment of estrogen and progesterone receptor status by immunohistochemical staining (positive ≥ 10% staining cells).

Prior to DNA extraction, the biopsies were analyzed with imprint cytology smears to confirm the presence of tumor cells. Imprint cytology smears were stained with the May–Grünwald–Giemsa stain (Color-Rapid, Med-Kjemi, Norway) and evaluated microscopically by a cytopathologist. The biopsies were analyzed using High-Resolution Magic Angle Spinning Magnetic Resonance spectroscopy (HR MAS MRS) prior to DNA extraction. This method is a nondestructive technique that provides the analysis of metabolites in intact tissue, and the same tissue was used for the subsequent DNA extraction. Results from metabolomics analysis have been described previously [18].

### DNA extraction and quality assessment

In total, 36 biopsies were excluded due to no available DNA (DNA isolation was not performed) and absence of tumor cells examined by imprint cytology. DNA was extracted from 130 biopsies (*n* = 55 pre-treatment samples, *n* = 75 post-treatment samples), and average weight of 15.1 mg ranges from 6.3 to 21.7 mg. After HR MAS MRS, the biopsies were homogenized using a rotor stator (max speed, 20 s). Genomic DNA was extracted using QIAGEN All prep DNA/RNA/protein isolation kit and quantified with NanoDrop™ 8000 Spectrophotometer (Thermo Fisher Scientific, Wilmington, DE, USA). DNA yielded was 7.2 ± 4.3 µg (average ± standard deviation), and 260/280 ratio was 1.9 ± 0.03. To examine the integrity of genomic DNA, a subset of samples (*n* = 24) was analyzed on a 2100 Bioanalyzer (Agilent Technologies, Santa Clara, CA, USA). Average peak size was 8661 bp (ranging from 3596 to 17,000 bp), and no samples showed sign of DNA degradation.

### DNA methylation analysis

DNA methylation was analyzed using the Illumina HumanMethylation450 BeadChip in 130 samples from 83 patients (*n* = 55 pre-treatment samples, *n* = 75 post-treatment samples). Paired pre- and post-treatment samples were available from 47 of the patients, but all 130 samples from the 83 patients were included in the analysis by using a linear mixed model, thereby increasing the power of the analysis. DNA methylation raw data were filtered with a site detection *p* value of < 0.05, and sites with more than 75% of measurements above the detection p value were removed from the analysis. Further the data were peak corrected to avoid type II probe bias as described by Dedeurwaerder et al. [32] and quantile normalized using the methy450PP function in the *minfi R* package [33].

DNA methylation from normal-adjacent breast tissue was downloaded from the TCGA data portal, *n* = 97 [34]. To assess whether the alterations in DNA methylation during treatment made the tumor samples more similar to histopathologically normal breast tissue, we compared the direction of alteration during treatment with the mean methylation of the normal breast samples, and determined the ratio of CpGs becoming more similar to normal breast tissue.

### Differentially methylated sites

**Fig. 1:**
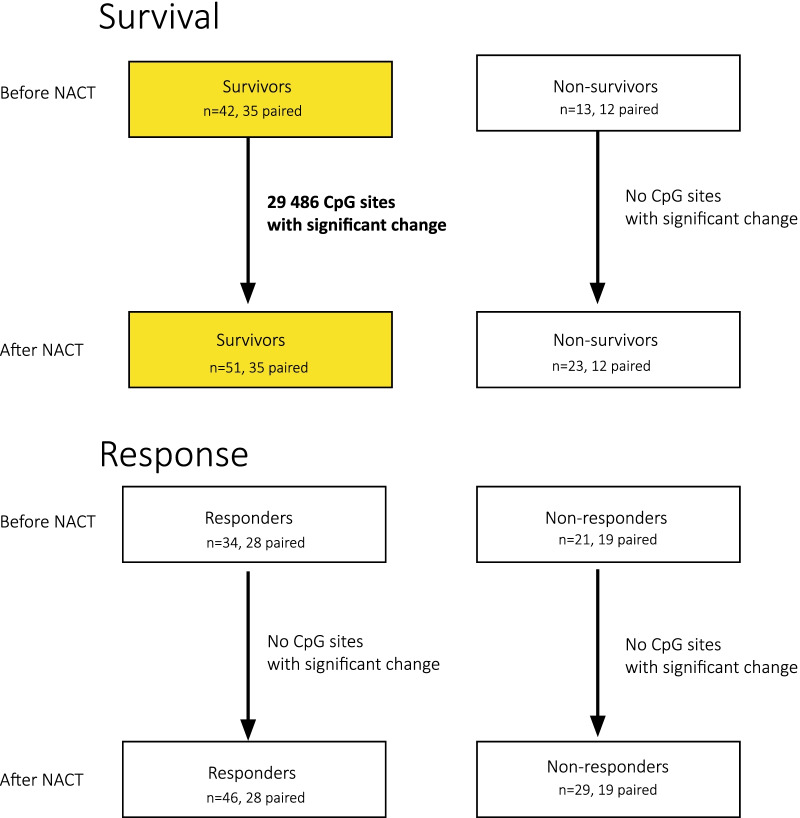
Comparisons of DNA methylation before and after NACT in different patient groups. The boxes represent groups compared in differential methylation analyses along with number of samples in each group. A statistically significant change from before to after NACT was found in 5-year survivors, and no significant changes were found in non-survivors, responders or non-responders

Differential methylation analysis was performed on the methylation M values in R version 4.0.5. The M values are the log transformed beta values *M* = *log2(beta/1-beta), where* beta is the ratio between the intensities of the methylated and unmethylated probes. The methylation M values before and after chemotherapy were compared in a linear mixed model using the R/Bioconductor package *limma* [35]. We compared samples before and after treatment in groups as shown in Fig. 1: 5-year survivors, 5-year non-survivors, treatment responders (i.e., patients with partial response) and treatment non-responders (i.e., patients with stable disease). We also compared samples before and after NACT in patients that received different chemotherapy regimens, and patients with different hormone receptor status and intrinsic subtypes. In all comparisons, we included patient ID using the function duplicateCorrelation in *limma*, to include it as a random effect, thus taking into account that some samples were from the same patient. P values were corrected for multiple testing using the Benjamini–Hochberg method [36]. Differentially methylated sites were defined as the probes with an adjusted *p* value of < 0.01.

### Pathway analysis

The locations of CpG sites related to CpG islands (CGIs) and genes were collected from the Illumina HM450k annotation, and the CpG sites with a statistically significant change in survivors during treatment were grouped into regions of interest (gene body CGI, gene body non-CGI, intergenic CGI, intergenic non-CGI, promoter CGI and promoter non-CGI). The proportion of differentially methylated sites was calculated as the number of statistically significant CpG sites in each group divided by the number of all the CpG sites in the Illumina array in the group. Pathway analysis was obtained by Gene Ontology and KEGG terms using the R package *missMethyl* [37]. Separate pathway analyses were performed on the CpG sites that had gained or lost methylation in the regions of interest. The top six terms in each analysis were plotted together with the -log10 of the false discovery rate (FDR) of overrepresentation. The relative immune cell fractions of the samples were calculated using the R package *MethylResolver* [38] with methylation beta values as input. MethylResolver estimates the relative fraction of immune cells in the sample using a reference cell methylation signature. The difference in immune cell fractions before and after treatment was compared using a Wilcoxon rank sum test.

### Methylation and prognosis

The differentially methylated sites were included in a LASSO (least absolute shrinkage and selection operator) regression model to further narrow the most important prognostic CpG sites for survival. The LASSO model was trained and validated with *leave-one-out* cross validation using the difference between the pre-treatment and post-treatment methylation beta values in the paired samples (47 patients, 94 samples) as covariates, and 5-year breast cancer-specific survival as the outcome using the R package *glmnet*. The CpG sites that had a nonzero coefficient in over 80% of the leave-one-out models were included in the final model, as well as the mean of the nonzero coefficients.

The risk score for each patient was calculated using the sum of the change in the methylation beta value in each CpG site from before to after treatment weighted by the coefficient from the LASSO model. The 10-year breast cancer-specific survival of the high- and low-risk groups was then plotted in a Kaplan–Meier plot. The high- and low-risk groups in the Kaplan–Meier are the risk group predicted for the left-out patient during the *leave-one-out* cross-validation.

### Validation cohort

We validated the risk score developed in this study in another previously generated data set [20, 39, 40]. The cohort consists of patients with locally advanced breast cancers that have received doxorubicin or a combination of 5-FU and mitomycin as NACT. The biopsies were analyzed using the Illumina Human Methylation 450 k BeadChip. Preprocessing and normalization involved steps of probe filtering, color bias correction, background subtraction and subset quantile normalization as previously described [41]. DNA methylation profiles from paired pre- and post-treatment samples were available for 85 patients, and the risk score was applied to the difference in methylation beta value from before to after treatment. The median of the risk score was used as cutoff between high and low risk. The breast cancer-specific survival of the risk groups was analyzed using a Kaplan–Meier plot.

## Results

### Differentially methylated CpG sites

We identified 29,486 differentially methylated sites when comparing the methylation of the samples before and after NACT in 5-year survivors (adjusted *p* value < 0.01), as shown in Fig. 1. The relation between the adjusted p value and effect size is shown in a volcano plot in Additional file 1: Fig. S1. There were no significant differentially methylated sites observed in 5-year non-survivors before versus after NACT, and the highest ranked sites by *p* value and –log fold change in survivors were low ranked in non-survivors (Additional file 1: Fig. S2). Furthermore, there were no significant changes in methylation sites from before to after NACT in either responders or non-responders (Fig. 1), hormone receptor positive, HER2 positive or within specific intrinsic subgroups (Additional file 1: Fig. S2). No significant difference in methylation was observed in patients that received different treatment regimens with epirubicin and/or paclitaxel when comparing samples before treatment and between each treatment group, samples after treatment between each treatment groups, as well as within each treatment group (before vs after NACT) (Additional file 1: Fig. S2). Further analyses were performed on the differentially methylated sites in 5-year survivors from before to after NACT. A complete list of the differentially methylated sites can be found in Additional file 3.

To investigate whether NACT-induced changes in tumor DNA methylation occurred in the direction toward or away from normal DNA methylation of normal breast tissue, we compared the mean of the 29,486 CpGs altered during treatment in our cohort with the methylation levels of normal-adjacent breast tissue from the TCGA cohort (*n* = 97). Of the 29,486 CpGs, 22,271 CpGs (90%) had a change in the direction of normal DNA methylation (density distribution shown in Additional file 1: Fig. S3).

### DNA methylation changes in different genomic regions in 5-year survivors

**Fig. 2:**
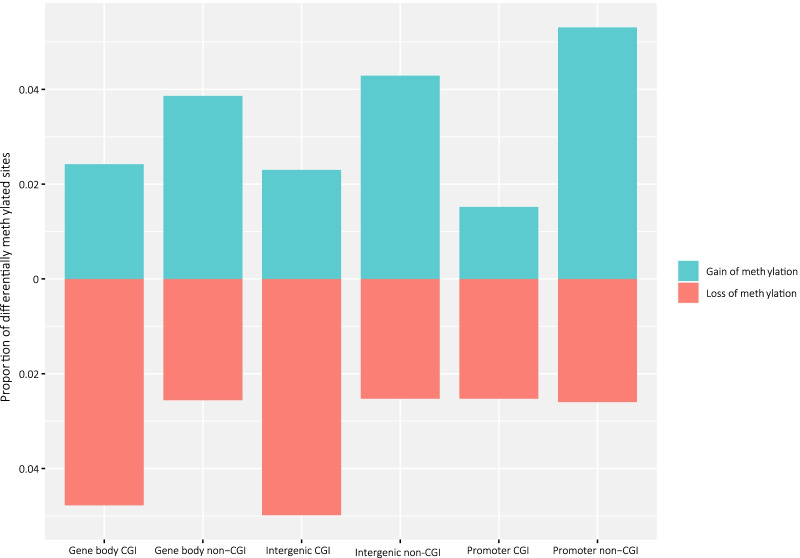
Differentially methylated sites by different regions in the genome. Most CpG sites with loss of methylation (red) are in gene body CpG islands (CGIs) and intergenic CGIs, while CpG sites that gained methylation (blue) after treatment are predominantly in non-CGI regions. CGI = CpG island

DNA methylation changes may have different effects on transcription and chromatin structure depending on their location in the genome. Therefore, we investigated the relative amount of differentially methylated CpG sites in regions of the genome to the total amount of CpG sites in the array in that region before versus after NACT in 5-year survivors (Fig. 2). The majority of differentially methylated CpG sites that lost methylation after treatment were in CpG islands, especially in gene body CpG islands and intergenic CpG islands. Most of the CpG sites that gained methylation after treatment were outside of CpG islands (non-CGI).

### Pathway analysis of differentially methylated sites in 5-year survivors

**Fig. 3:**
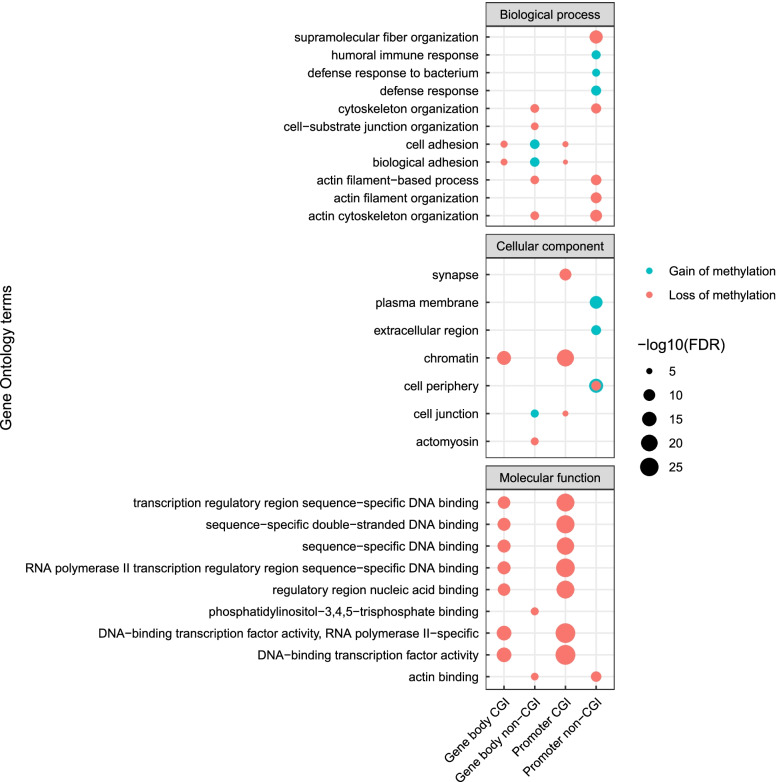
Pathway analysis of the differentially methylated CpG sites in the four genomic regions with significant findings (gene body CGIs, gene body non-CGIs, promoter CGIs and promoter non-CGIs) in 5-year survivors. Sites with loss of methylation are predominantly enriched for terms related to sequence-specific DNA binding, cell adhesion and the plasma membrane, while many of the sites with a gain in methylation are enriched for terms related to the immune response. The size of the dots represents the -log10 of the FDR of overrepresentation, and the color indicates if the sites related to the term had gained (blue) or lost (red) methylation. *CGI* = *CpG island*

We investigated the biological implications of the differentially methylated sites in each region in the genome by performing separate pathway analyses on the CpG sites in each region (Fig. 3). The main observation is the consistent loss of CpG island methylation in genes related to sequence-specific DNA binding and transcription factor activity (Fig. 3, molecular function). This loss of methylation was most prevalent in promoters, but also occurred in gene body and intergenic CpG islands. In addition, loss of CpG island methylation in promoters and gene body also affected genes involved in cell adhesion (Fig. 3, biological process) and the plasma membrane (Fig. 3, cellular component). In contrast, gain of methylation was most observed in promoters not situated in CpG islands and genes regulating immune response, cell adhesion and the plasma membrane. There were no enriched terms in intergenic regions.

**Fig. 4:**
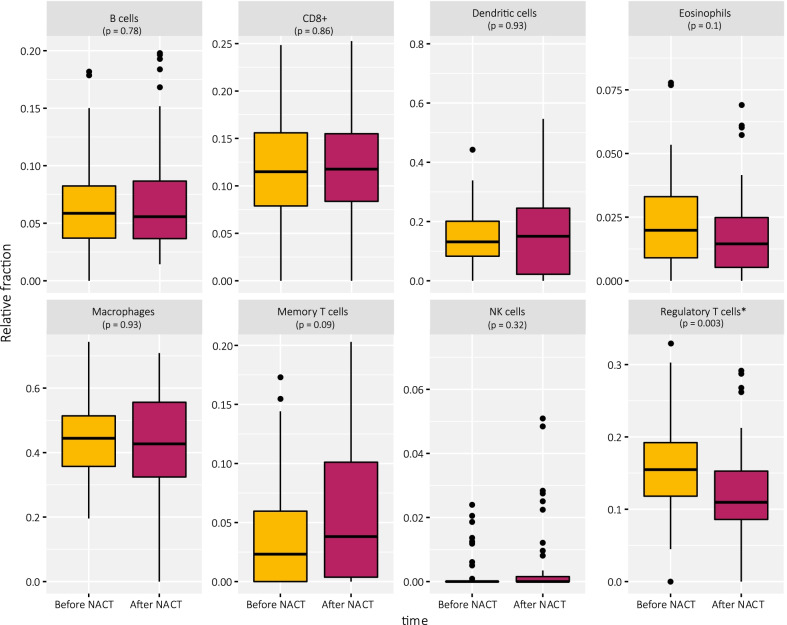
Estimated relative fractions of immune cells in the tumor samples before and after treatment in 5-year survivors. There was a significant decrease in regulatory T cells in survivors after NACT (*p* = 0.003), *p < *0.05*

As gene ontology analysis showed a gain of methylation in immune response genes after NACT, we further estimated the relative immune cell fractions in pre- and post-treatment samples of survivors and non-survivors. The estimated fractions of regulatory T cells were significantly lower post-treatment in survivors (*p* = 0.003, Fig. 4), while there were no significant differences in the other immune cells from before to after treatment. No statistically significant difference in immune cell composition was found in non-survivors from before to after NACT (Additional file 1: Fig. S4), and there were no significant differences between 5-year survivors and 5-year non-survivors before NACT.

### DNA methylation change and breast cancer prognosis

**Fig. 5:**
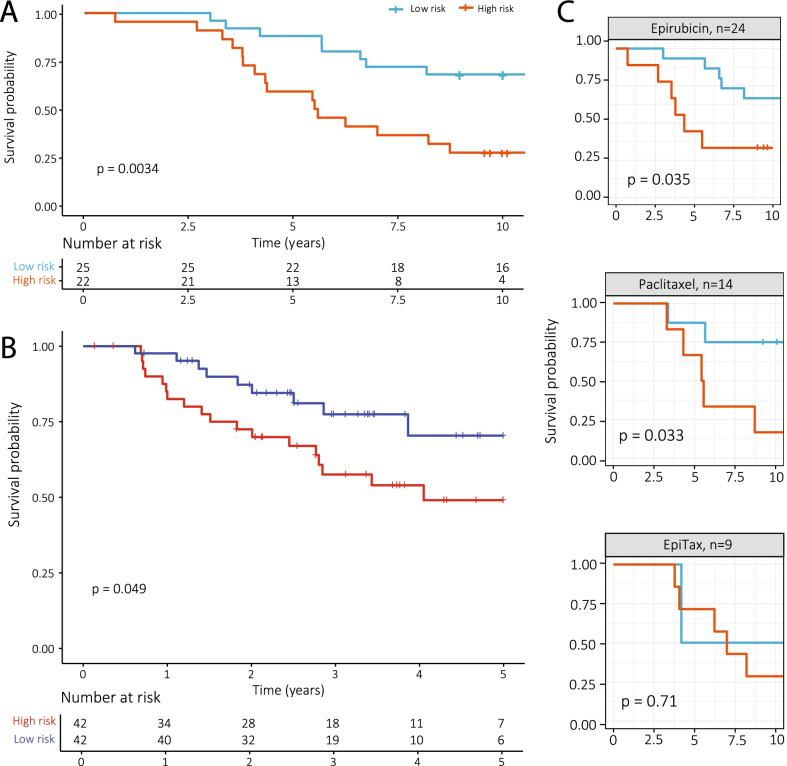
Kaplan–Meier plot showing the breast cancer-specific survival of the methylation risk groups based on changes in methylation from before to after NACT. **A** The methylation risk score separated the risk groups significantly (*p* value = 0.0034) in our cohort. **B** The risk score was validated in an independent cohort of locally advanced breast cancer patients receiving NACT (*p* value = 0.049). **C** The risk score separated the risk groups significantly in patients receiving monotherapy epirubicin (*p* = 0.035) and paclitaxel (*p* = 0.033), but not a combination of the two. *EpiTax: epirubicin followed by paclitaxel in case of inferior response or paclitaxel followed by epirubicin in case of inferior response*

We investigated if the changes in methylation from before to after NACT could predict the survival of breast cancer patients. We developed a DNA methylation risk score using the difference between pre-treatment methylation and post-treatment methylation of the differentially methylated sites observed in survivors (*n* = 29,486) in a LASSO regression model. After *leave-one-out cross-validation*, we identified four CpG sites with nonzero coefficients, and the risk score was constructed using these sites and the mean of the coefficient from each model in the cross-validation. The survival of the risk groups from the LASSO regression is shown in Fig. 5A, where the risk score is applied to the change in methylation for each of the patients with both pre-treatment and post-treatment samples (47 patients). The risk score with probe names and coefficients is given in Additional file 2.

In our patient cohort, the median survival in the high-risk group was 5.53 years, while median survival in the low-risk group was at least ten years. In the high-risk group, 59% of the patients were alive at 5 years and 18% at 10 years. In contrast, in the low-risk group 5- and 10-year survival was 88% and 64%, respectively. The risk score developed based on our cohort was applied in an independent cohort of locally advanced breast cancer patients receiving NACT, showing significantly different survival curves based on changes in methylation also in this cohort (*p* = 0.049, Fig. 5B).

When examining treatment groups separately, survival distributions were significantly different in the patient groups receiving monotherapy regimens, but not in the group receiving first paclitaxel followed by epirubicin or vice versa, although this could be due to a low patient number in this group (only 2 patients receiving EpiTax were classified as low risk) (Fig. 5C). When analyzing the treatment groups separately in the validation cohort, we observed survival difference in the patients treated with 5-FU and mitomycin C, but no survival difference in the patients treated with doxorubicin (Additional file 1: Fig. S5).

## Discussion

In this study, we detected significant changes in DNA methylation in 5-year breast cancer survivors when comparing biopsies taken before and after NACT. We further developed a risk score based on methylation sites with significant change before versus after NACT associated with 5-year survival in our patient cohort that was validated in an independent cohort. While a few previous studies have described DNA methylation changes from before to after treatment in patients with breast cancer [20, 23], this is the first study to correlate methylation changes to survival. In our study, the methylation changes were unique to survivors, as there were no significant changes in CpG sites in non-survivors from before to after treatment. Additionally, the top-ranked CpG sites by p value and effect size in survivors were low ranked in non-survivors, indicating that the difference in results between survivors and non-survivors is not due to differences in sample size.

The prognostic value of the risk score developed in this study was validated in a separate cohort of locally advanced breast cancer patients. This supports the idea that the changes in methylation during neoadjuvant treatment are important for breast cancer survival. The validation cohort had a similar treatment regimen as our discovery cohort, since epirubicin and doxorubicin are similar drugs. However, since a proportion of patients in both cohorts received other treatment regimens (paclitaxel or 5-FU and mitomycin) and the separation of the risk groups receiving different treatments in both cohorts were significant, this suggest that the risk score is not specific for a given NACT and may be valid independently of treatment regimen. Our study shows that changes in tumor DNA methylation are associated with survival in two separate cohorts. This highlights the importance of studying the molecular response of breast cancer tumors during NACT to be able to assist the prognosis of breast cancer patients. Although the risk score could not be used to predict survival before treatment, assessment of prognosis post-treatment would be useful to pinpoint the patients in need of closer follow-up and possibly extended treatment.

Given the complex association between DNA methylation and gene expression, it can be difficult to assess the exact function of single methylation sites. By investigation of the close by genes or the genes previously associated with the CpGs, we tried to interpret the biological relevance of the CpG sites in the methylation risk score. The first differentially methylated site (cg10298059) is annotated to the gene body of ZFHX3, which is a transcription factor and tumor suppressor gene, its expression associated with the prognosis of breast cancer patients [42–44]. The second CpG site (cg27034150) is in the promoter region of SULT1A1, which is a sulfotransferase involved in the metabolism of drugs. Its involvement in tamoxifen metabolism has been reported previously [45–47]. The third CpG site (cg01503450) is situated in the promoter region of LARP4B, a gene involved in RNA translation, and could be a tumor suppressor gene [48–50]. The fourth CpG site (cg07959469) is situated downstream of the gene NR2F2, which is a transcription factor involved in ER-mediated transcriptional regulation and also involved in invasion and migration [51–53]. In summary, the four identified CpGs are located around genes important for cancer cells and may explain why alterations in DNA methylation at these CpGs are associated with prognosis.

CpG islands in normal cells are in general unmethylated, but during aging and cancer development, there is an overall gain of methylation in CpG islands, while the rest of the genome loses methylation [10, 54]. Gain of methylation in CpG islands can repress tumor suppressor, apoptosis, cell adhesion and DNA reparation genes, while loss of methylation outside CpG islands is associated with activation of oncogenes, reactivation of fetal genes and loss of repression of transposable elements, leading to chromosomal instability [55, 56]. In our study, there was a predominant loss of methylation in CpG islands in 5-year survivors after treatment and an overall gain in methylation outside of CpG islands. In addition, 90% of the differentially methylated sites had a change in methylation toward normal breast tissue. The combined methylation changes we observed for the survivors in our study thus suggest a reverse cancer progression or fewer aggressive cells in the tumor after treatment.

When exploring the biological implications of the differentially methylated sites in functional regions of the genome, we found reduced methylation of CpG islands in genes involved in transcription factor activity and cell adhesion. Differential methylation associated with these biological processes has also been found to discriminate breast cancer from normal tissue [57] and to be associated with response to NACT in triple-negative breast cancer [58]. DNA methylation is known to regulate transcription factors in human cancers, which in turn regulates oncogenes and signaling pathways important for prognosis [9, 16, 59]. Cell adhesion genes are less methylated in noninvasive breast cancer cell lines compared to invasive breast cancer cell lines [60]; thus, a loss of methylation as observed in this study could be a sign of less invasive cancer cells in the post-treatment samples. Since the relation between DNA methylation and gene expression is complex, it is important to validate these findings by gene expression and protein analysis before developing therapeutics targeting these processes.

Methylation of immune response genes before treatment in breast cancer has previously been associated with prognosis [61] as well as the infiltration of lymphocytes in the tumor [62]. Since we found immune system genes overrepresented in the pathway analysis, we explored this further by immune cell deconvolution by MethylResolver [38] and found a reduced estimated T cell fraction in the samples after treatment.

The function of regulatory T cells is to regulate and moderate immune reactions. In the tumor microenvironment, they are pro-tumorigenic and protect the tumor from immune destruction [63]. Thus, fewer regulatory T cells make the tumor more exposed to the anti-tumor immune response. Low regulatory T cell infiltration identified by immunohistochemistry has been connected to a better prognosis (complete response) in locally advanced and local breast cancer [64–67]. In this study, 5-year survivors and non-survivors had no significant differences in immune cell composition before treatment. However, the survivors’ immune cell composition changed toward a more anti-tumorigenic response, highlighting that the dynamics of the immune infiltration could be important for patient survival, especially as a response to chemotherapy. Although the effect of treatment-induced immune response observed in our study is interesting, the immune response is a complex system and additional studies and methods are needed to investigate this in more details.

In previous studies, both survival and DNA methylation patterns have been related to the molecular subgrouping and receptor status of breast cancer [14], and breast cancer may even be further sub-grouped using DNA methylation and clustering methods [12, 68, 69]. It is therefore important to establish whether prognostic biomarkers are valid in one, several or across all subgroups. As there were no significant changes in methylation in hormone receptor positive, HER2 positive or within specific intrinsic subgroups from before to after NACT, we conclude that the change in methylation is either consistent across subtypes or that there are too few patients in each subgroup to detect subtype-specific differential methylation.

Studies using DNA methylation signatures to predict NACT response have previously been conducted in pre-treatment tumor biopsies and blood samples of breast cancer patients [21, 22, 58]. However, we did not find DNA methylation changes that were related to treatment response. A plausible reason could be because our study cohort does not include patients with complete response where no residual tumor was left after treatment, nor patients with progressive disease. In addition, the evaluation of treatment response was performed based on caliper measurement of the tumor before and after treatment, which could heighten the risk of inaccurate measurements, as compared to radiological evaluations. Also, the treatment response criteria used in our study were according to the UICC recommendation applicable at the time of patient recruitment, and some of the patients with stable disease would have been classified as partial responders according to the RECIST criteria used today. In the current study, 5-year non-survivors had a lower percentage of partial response compared to 5-year survivors, 45.8% versus 69.6%, respectively. In our previous study of the same patient cohort, we detected changes in tumor metabolism after treatment. The changes were related to survival but not to treatment response [18], similar to what we observed for methylation patterns in the current study. Many patients in the non-survival group had a relatively good response to treatment, but still experienced a rapid progression. This implies the importance of studying the molecular tumor response to treatment and its effect on survival in addition to shrinkage of tumor size.

The patients included in this study were treated with monotherapy regimens consisting of either epirubicin or paclitaxel. In case of non-satisfactory response, they were assigned to the other chemotherapy. Although standard clinical guidelines today advocate a combination of different chemotherapies, our study investigates the effect of these two drugs both separately and in sequence. Our results show no significant differences in DNA methylation when comparing the different treatment regimens, which suggest that differential DNA methylation observed in survivors is not dictated by either of these two chemotherapies initiated as the first regimen or when given in sequence. Interestingly, the risk score could separate patients having received monotherapy by either epirubicin or paclitaxel into low- and high-risk groups when the treatment groups were examined separately.

This study has some limitations. The cohort contains a mix of breast cancer subtypes, which introduced challenges due to heterogeneity. The patients were treated with monotherapy, which is an older treatment regimen compared to nowadays. The study is constrained by the lack of available tumor tissues for further analyses, especially in case of validation of estimated immune cell fractions, for example, by immune histochemistry or quantitative polymerase chain reaction (qPCR). However, the signature developed has been validated in an independent cohort, which demonstrates its clinical potential with regard to survival. Further studies are, however, needed to fully understand the biological implication of these methylation sites and how they are associated with breast cancer prognosis.

## Conclusion

In this study, we demonstrate changes in DNA methylation patterns from before to after NACT in 5-year survivors of locally advanced breast cancer. We developed a risk score consisting of four CpG sites that could predict long-term survival in our patient cohort and a separate validation cohort. Our results provide novel biological insight to how tumors respond to treatment and suggest that DNA methylation analysis could be used as prognostic tool to predict survival outcome in breast cancer patients treated with neoadjuvant chemotherapy.

## Supplementary Information

**Additional file 1.** A document displaying supplementary figures.

**Additional file 2.** A table showing the four-CpG DNA methylation risk score created by the leave-one out LASSO model and their coefficients.

**Additional file 3.** A list of the significant CpG sites before vs after treatment in survivors, along with the p value and Illumina annotation for each site.

## Data Availability

The data that support the findings of this study are not openly available due to sensitivity of human data but are available from the corresponding author upon request.

## Abbreviations

NACT: Neoadjuvant chemotherapy
CGI: CpG island
CpG: Cytosine–phosphate–guanine dinucleotide
