# Supplementary material for: DNA methylation changes in response to neoadjuvant chemotherapy are associated with breast cancer survival
Source: Breast Cancer Res. 2022 Jun 24;24:43. doi: 10.1186/s13058-022-01537-9 (PMC9233373; doi:10.1186/s13058-022-01537-9)
Supplement: Supplementary file 1 — Additional file 1. A document displaying supplementary figures. [file 13058_2022_1537_MOESM1_ESM.docx]

**Additional file 1: Supplementary figures**


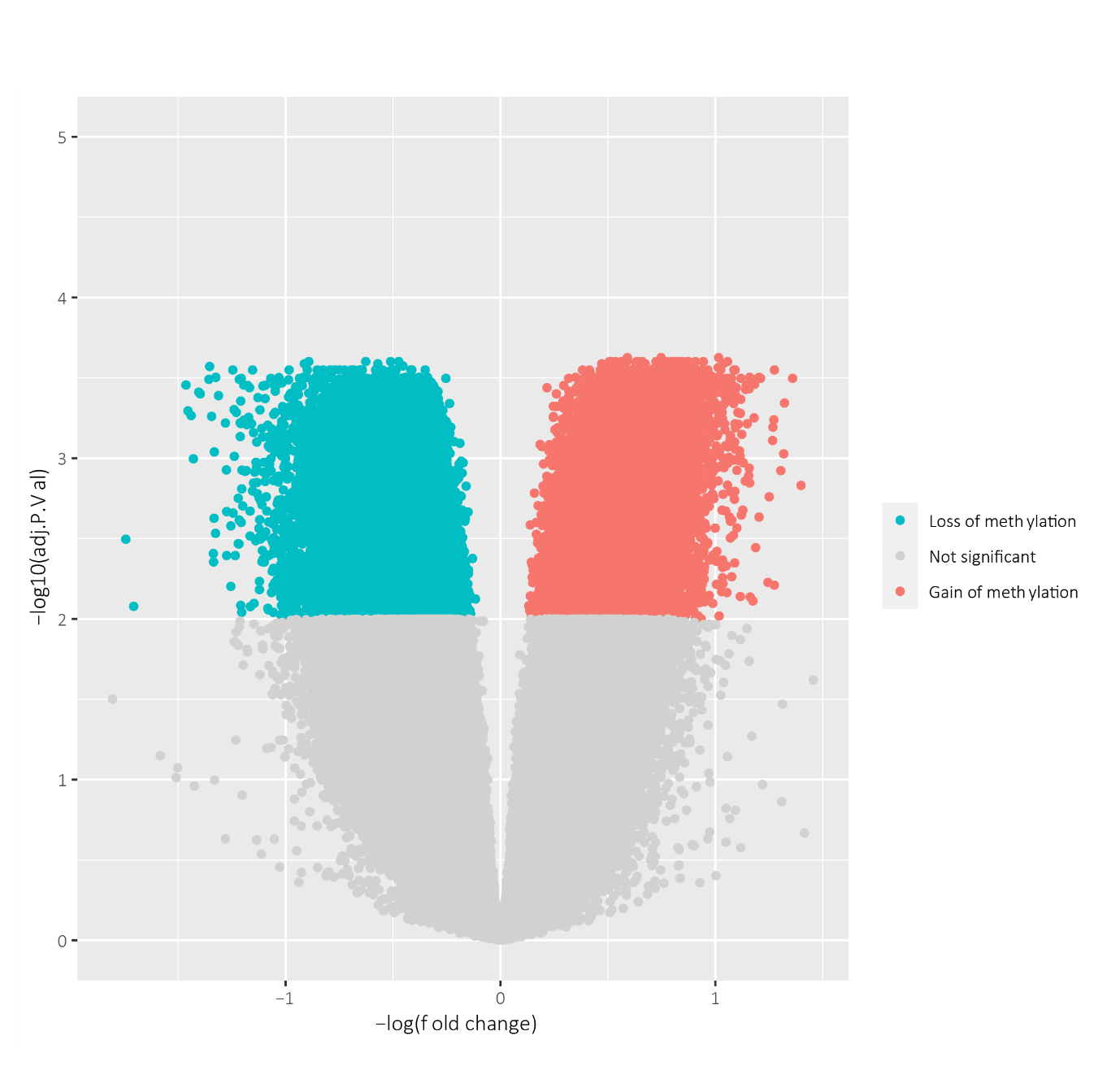
Supplementary Figure 1: Volcano plot of the differences in DNA methylation sites in 5-year survivors before and after NACT. The dots represent each tested CpG site with the -log10 of the FDR-adjusted p-value relative to the effect size (log10 fold change). The statistically significant CpG sites that gained methylation after treatment are plotted in blue, and the statistically significant sites that were less methylated after treatment are plotted in red.


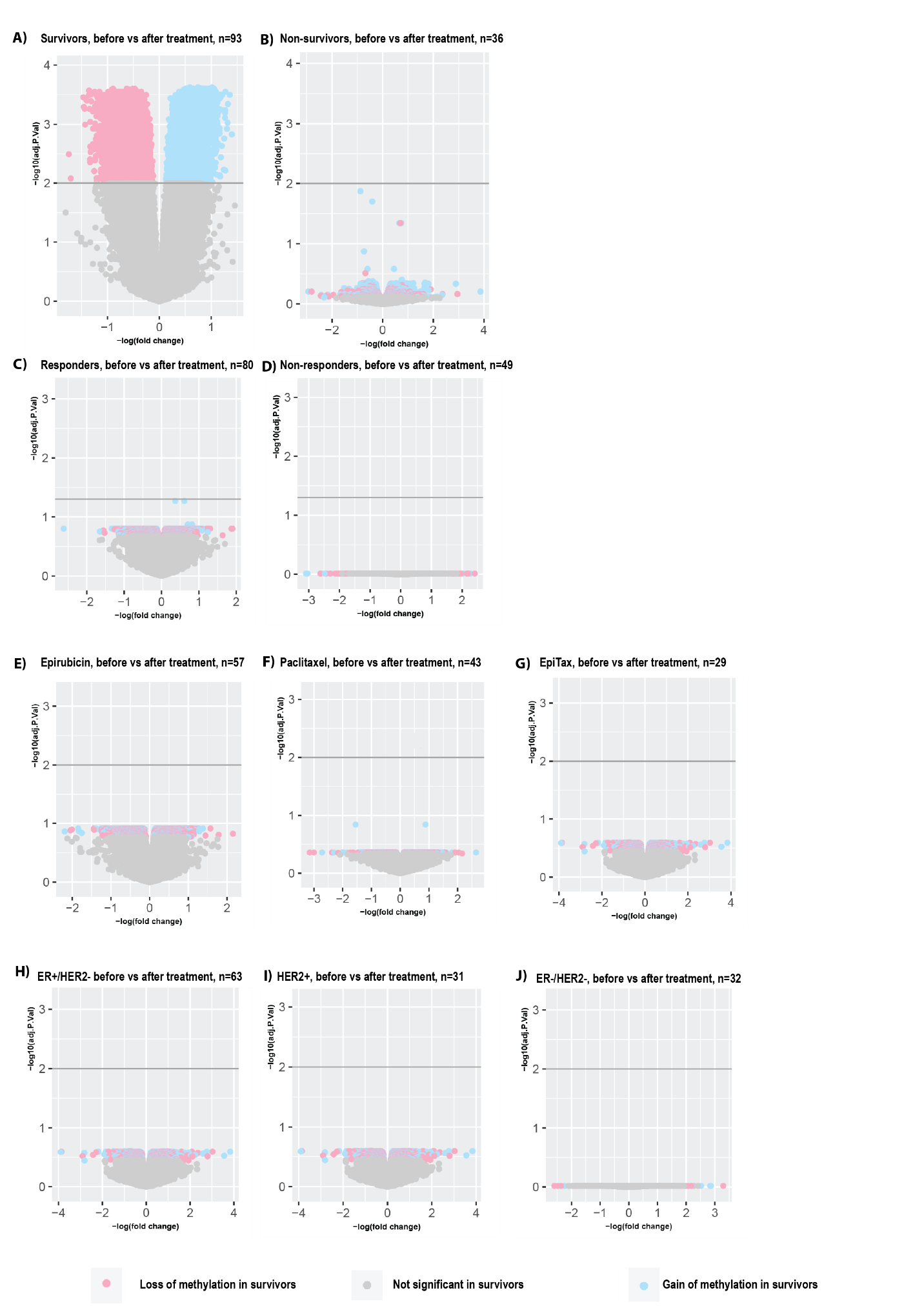


Supplementary figure 2: Supplementary Figure 2: Volcano plots of DNA methylation sites stratified by survival, treatment response, treatment regimens and intrinsic subtypes. The dots indicate loss (pink) and gain (blue) of DNA methylation observed in survivors (before vs after treatment, in total 29,486 significant DNA methylation sites). A) Survivors (same figure as Supplementary Figure 1), B) Non-survivors, C) Responders, D) Non-responders, E) Monotherapy epirubicin, F) Monotherapy paclitaxel, G) Epirubicin followed by paclitaxel or vice versa, H) ER positive, HER2 negative, I) HER2 positive and J) ER negative, HER2 negative.


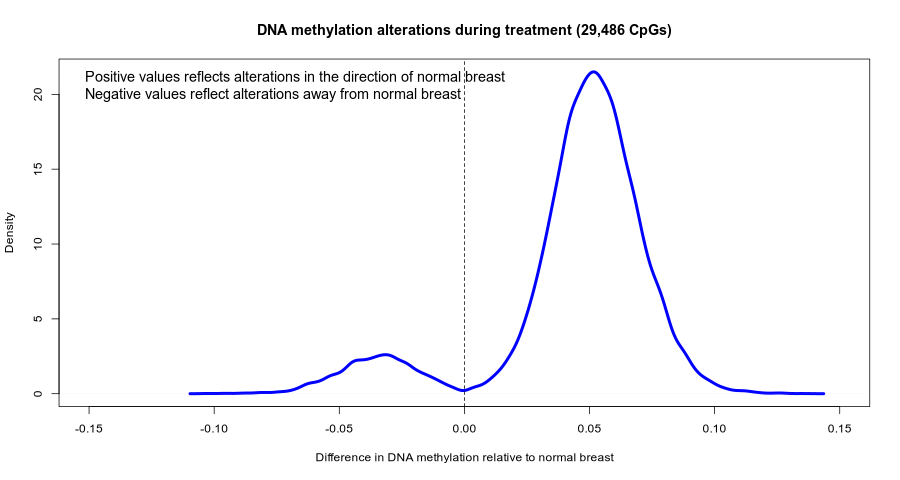
Supplementary Figure 3: To assess whether the alterations in DNA methylation during treatment made the tumor samples more similar to normal breast, we leveraged the normal adjacent breast tissue from the TCGA cohort (n=97). The mean of the 29,486 CpGs altered during treatment was calculated across the 97 normal adjacent samples, and we determined whether the treatment induced changes in tumor DNA methylation was in the direction towards or away from normal DNA methylation. Of the 29,486 CpGs, 22,271 CpGs (90%) had a change in the direction of normal DNA methylation.


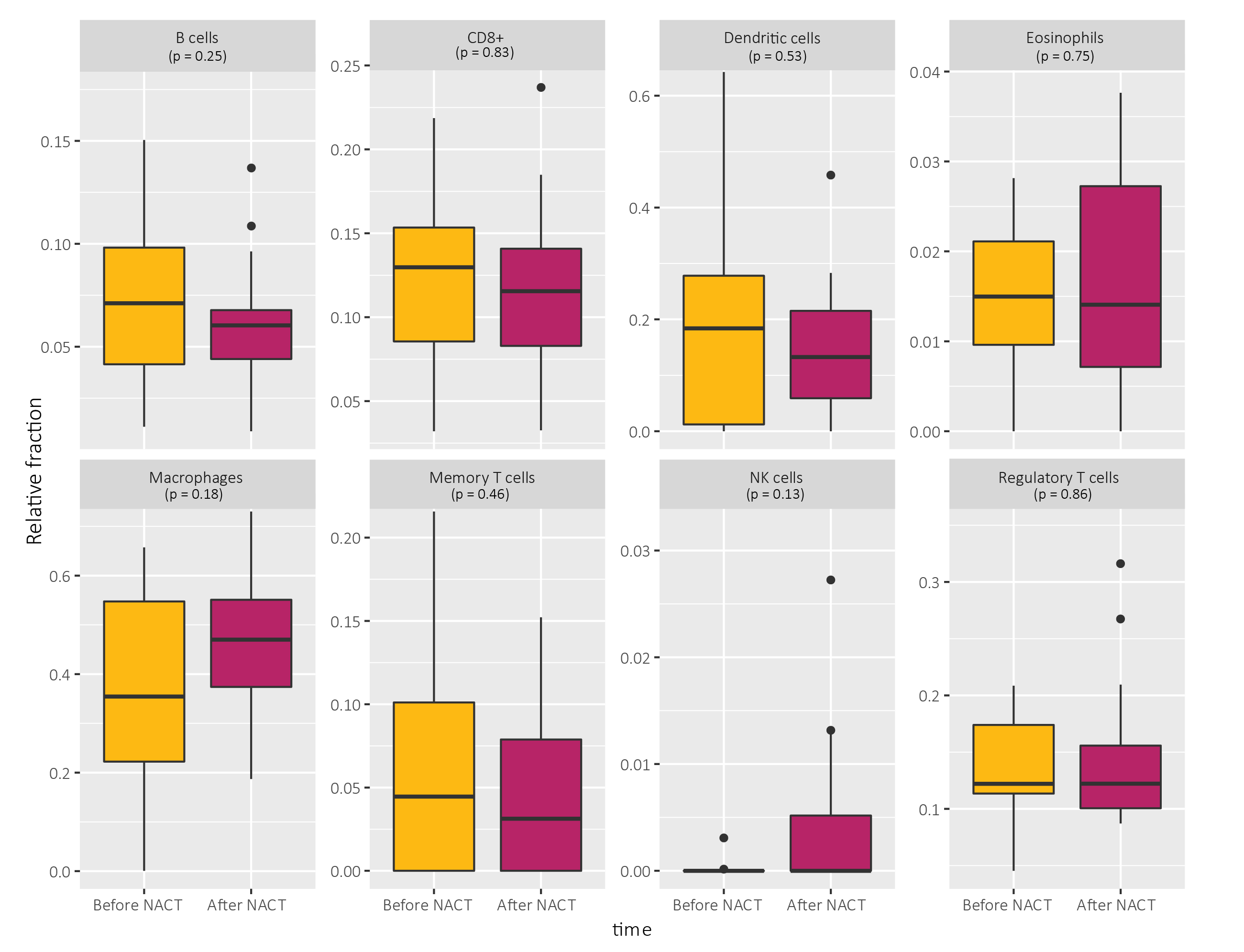


Supplementary Figure 4: Estimated relative fractions of immune cells in the tumor samples before and after treatment in 5-year non-survivors. There were no significant changes in immune cell composition.


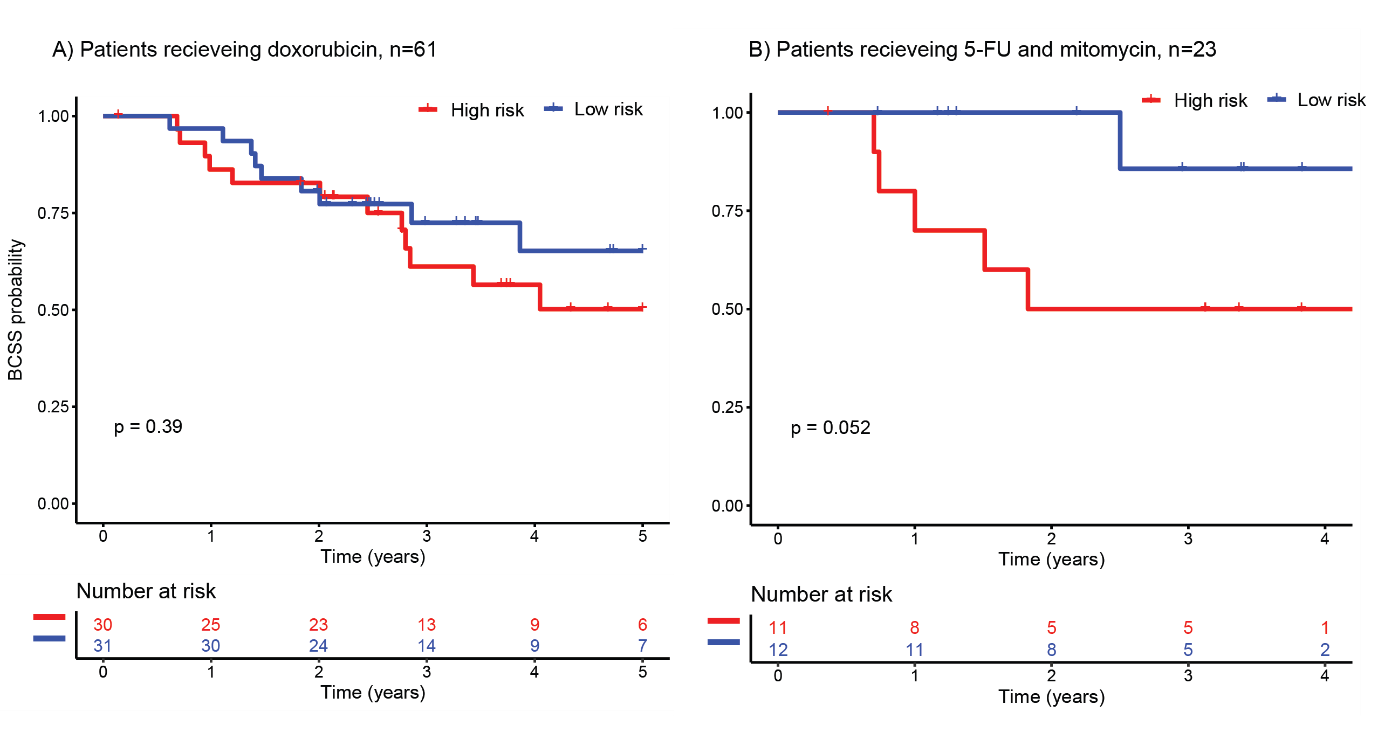
Supplementary figure 5: Survival plots of the high and low risk groups in the validation cohort. A) The separation between high and low risk groups in patients that received doxorubicin monotherapy was not significant (p=0.39) B) There is a significant separation between high and low risk groups in patients that received 5-FU and mitomycin (p=0.052)
